# Towards understanding the drivers of policy change: a case study of infection control policies for multi-drug resistant tuberculosis in South Africa

**DOI:** 10.1186/s12961-017-0203-y

**Published:** 2017-05-30

**Authors:** Trust Saidi, Faatiema Salie, Tania S. Douglas

**Affiliations:** Division of Biomedical Engineering, Department of Human Biology, University of Cape Town, P. Bag X3, Observatory, 7935 Cape Town, South Africa

**Keywords:** Multi-drug resistant tuberculosis, Policy changes, Infection control, Policy streams

## Abstract

**Background:**

Explaining policy change is one of the central tasks of contemporary policy analysis. In this article, we examine the changes in infection control policies for multi-drug resistant tuberculosis (MDR-TB) in South Africa from the time the country made the transition to democracy in 1994, until 2015. We focus on MDR-TB infection control and refer to decentralised management as a form of infection control. Using Kingdon’s theoretical framework of policy streams, we explore the temporal ordering of policy framework changes. We also consider the role of research in motivating policy changes.

**Methods:**

Policy documents addressing MDR-TB in South Africa over the period 1994 to 2014 were extracted. Literature on MDR-TB infection control in South Africa was extracted from PubMed using key search terms. The documents were analysed to identify the changes that occurred and the factors driving them.

**Results:**

During the period under study, five different policy frameworks were implemented. The policies were meant to address the overwhelming challenge of MDR-TB in South Africa, contextualised by high prevalence of HIV infection, that threatened to undermine public health programmes and the success of antiretroviral therapy rollouts. Policy changes in MDR-TB infection control were supported by research evidence and driven by the high incidence and complexity of the disease, increasing levels of dissatisfaction among patients, challenges of physical, human and financial resources in public hospitals, and the ideologies of the political leadership. Activists and people living with HIV played an important role in highlighting the importance of MDR-TB as well as exerting pressure on policymakers, while the mass media drew public attention to infection control as both a cause of and a solution to MDR-TB.

**Conclusion:**

The critical factors for policy change for infection control of MDR-TB in South Africa were rooted in the socioeconomic and political environment, were supported by extensive research, and can be framed using Kingdon’s policy streams approach as an interplay of the problem of the disease, political forces that prevailed and alternative proposals.

## Background

Multidrug-resistant tuberculosis (MDR-TB) is a major health problem affecting hundreds of thousands of people annually worldwide [1, 2]. Globally, the incidence of MDR-TB is increasing, with 480,000 new cases of MDR-TB and an estimated 190,000 TB-related deaths in 2014 [3]. South Africa is one of 22 high-burden countries that account for the majority of incident MDR-TB cases [1] and has the second-largest number of diagnosed MDR-TB cases [4]. It is estimated that approximately 1.8% of new TB cases and 6.7% of previously treated TB cases in South Africa are MDR [4, 5]. According to a report published by the Department of Health [6], approximately 450,000 new cases of TB occurred in South Africa in 2013, including more than 26,000 cases of MDR-TB. This is burdensome as MDR-TB is extremely expensive to treat, requiring ZAR 25,000–30,000 per patient for the drugs alone as opposed to less than ZAR 200 for a new patient with ordinary TB [7]. The MDR-TB epidemic in South Africa is worsened by the HIV epidemic as a result of the impaired ability of the immune system to contain TB bacilli and inadequate airborne infection prevention and control (IPC) measures [8–11]. The high prevalence of MDR-TB in South Africa underscores the importance of effective infection control and treatment programmes [12–14]. MDR-TB infection control remains one of the major cornerstones underpinning TB management programmes [15, 16].

A 2006 surveillance study of drug-resistant TB in patients with suspected or diagnosed TB in a rural resource-limited setting in South Africa with a high prevalence of HIV found a substantially higher prevalence of extensively drug-resistant (XDR)- and MDR-TB than previously reported [17]. The study raised concerns about the probability of nosocomial transmission of XDR-TB for two reasons. First, the majority (55%) of patients had not previously received TB treatment, and a further 30% had previous outcomes of cured or completed treatment. Genotyping showed that their strains were similar, and there was a possibility that transmission of strains occurred amongst these individuals. Second, two-thirds of the XDR-TB patients had been hospitalised at the same district hospital in the 2 years prior to their diagnosis. The possibility of nosocomial transmission of TB triggered responses to minimise the transmission of TB through IPC measures.

Against this background, this paper focuses on the policy perspectives on the accommodation of MDR-TB patients and measures for infection control in South Africa. Several studies have addressed MDR-TB IPC in South Africa. Notably, Farley et al. [13] evaluated national infection control strategies, and provided an overview of IPC measures, but did not cover policy aspects. The results of their study revealed that IPC measures were generally poorly implemented in the majority MDR-TB hospitals. To our knowledge, the IPC policy framework and its implications for MDR-TB have not been not addressed in the literature.

The incorporation of IPC practices may interrupt the transmission of TB in healthcare facilities. The WHO and the Centres for Disease Control and Prevention have produced IPC guidelines for airborne infectious diseases such as TB. These guidelines have been adapted to address resource-limited settings [18, 19]. All of these proposed guidelines present a hierarchy of control measures, which are:

1. Administrative control measures to prevent the generation of and/or exposure to droplet nuclei, thereby reducing exposure to the bacteria. These include promptly identifying people with TB symptoms (triage), separating infectious patients, controlling the spread of pathogens (cough etiquette and respiratory hygiene) and minimising time spent in healthcare facilities.
2. Environmental control measures for high-risk areas, which either remove the bacteria from the air or reduce the concentration of bacteria in the air.
3. Personal protective equipment used by exposed individuals to protect themselves from inhaling contaminated air, e.g. the wearing of respirators.

IPC measures are especially important for the management of MDR-TB given the challenges associated with its diagnosis and treatment [20, 21]. They are designed to protect not only patients, but also those who work in healthcare settings as they are at higher risk of becoming infected with MDR-TB [22]. The risks of MDR-TB transmission in healthcare and congregate settings underline the urgent need to refocus attention on IPC measures [23–25].

In order to systematically assess the evolution of policy on IPC measures and its significance for MDR-TB in South Africa, we used Kingdon’s theory of policy streams [26] to examine the factors driving policy change. Kingdon’s framework is suitable for our analysis as it allows a differentiated examination of how certain ideas enter government agendas and influence policy change. We also consider the role of research evidence in driving policy change. We focus on the period from 2000, when the first policy on MDR-TB was introduced in South Africa to when the latest one was introduced in 2013. According to Sabatier and Jenkins-Smith [27], policy change needs to be observed over a decade or more for analysis of how policy shapes the agenda and learning takes place.

### Theoretical framework: Kingdon’s policy streams approach

Kingdon’s policy streams approach assumes continual policy change. All the elements of the policymaking process shift and change, and policy outcomes arise from the continual interplay [26]. In explaining the process of policy change, Kingdon distinguishes three streams, namely problem recognition, policy proposals and politics. Although these streams work largely independently of each other, they come together at certain critical points, at which the greatest policy change is most likely to happen.

In the stream of problem recognition, the central question is why governments pay attention to some problems and not others. Problems enter the agenda for policy change for various reasons. Often, problems tend to come to the attention of government decision-makers not through a given political pressure, but because certain relatively symbolic indicators simply show that a problem exists. Governments tend to monitor the performance of systems through standard indicators, often numerical, such as deaths, costs and disease rates. Sudden shifts in the indicators or performance contrary to expectations attract attention and demand policy changes [28]. However, problems are not self-evident from indicators. Instead, a focusing event like a crisis, disaster or personal experience or powerful symbol might draw attention to a problem. However, such events have only transient effects unless accompanied by a firmer indication of a problem, by a pre-existing perception, or by a combination with other similar events. Feedback given on a particular problem can set the impetus for policy change; such feedback may include programmes that are not working as planned and consequences of programme enactment.

In the stream of policy proposals, a policy community of specialists, including bureaucrats, people in planning, evaluation and budgeting, academics, interest groups and researchers, constantly generate proposals. Their ideas bubble around in the policy communities in what is termed a ‘policy primeval soup’ [26]. The development of proposals must be performed long before the opportunity for actual adoption presents itself [28]. The ideas and proposals are subject to a selection process, with some being taken into consideration, while others are discarded. Kingdon identifies three criteria that support the placement of policy proposals on a government’s short list, namely they have to be technically feasible, not too expensive, and acceptable to the public. In addition, the values held by the public can constrain the choices that are made in specialised communities.

For Kingdon’s third stream on politics, the political changes in the administration, partisan or ideological composition of parliament, and interest group pressure, are central factors influencing policy agenda setting and subsequent policy changes. People in and around government sense a national mood, the climate in the country, changes in public opinion and broad social movements. It is when a large number of people in the country are thinking along certain common lines that policy agendas and outcomes are bound to change. In addition, organised political forces in the form of interest group pressure and political mobilisation provide a powerful force for policy change.

Certain conditions favour agenda change leading to policy evolution. According to Kingdon [26], joining the streams requires policy entrepreneurs who are more or less constantly at work on pointing attention to particular problems or policies. However, their likelihood of influencing the policy agenda is enhanced by a policy window, which can open either because of change in the political stream (e.g. an administration change) or the prevailing mood among the public. Politicians decide to undertake some sort of initiative on a particular subject and cast about for ideas to mobilise support for their proposals and capitalise on the prevailing national mood. Putting themselves in the market for proposals creates a window for advocates, and many alternatives are then advanced by their sponsors. In some cases, policy change may occur in situations where both the problems and their solutions may not have changed at all. Instead, the availability of an alternative that somehow responds to a new political situation can change the policy agenda.

## Methods

The study is based on a review of relevant literature and an analysis of policy documents on MDR-TB infection control in South Africa. Policy documents were extracted for the period 1994 to 2014, with a focus on infection control policies and not on treatment of MDR-TB. Five policy documents were identified on the basis that they addressed MDR-TB. To complement the policy documents, literature on TB infection control and MDR-TB in South Africa was reviewed. The literature search comprised a 20-year review of PubMed (1994–2014), but commonly cited and highly regarded new and older publications were not excluded. We searched focusing on “multidrug-resistant tuberculosis”, “MDR-TB”, “infection control” and “South Africa” on PubMed using the search terms:

(((“tuberculosis, multidrug-resistant”[MeSH Terms] OR (“tuberculosis”[All Fields] AND “multidrug-resistant”[All Fields]) OR “multidrug-resistant tuberculosis”[All Fields] OR (“multi”[All Fields] AND “drug”[All Fields] AND “resistant”[All Fields] AND “tuberculosis”[All Fields]) OR “multi drug resistant tuberculosis”[All Fields]) OR ((“microb drug resist”[Journal] OR “MDR”[All Fields]) AND TB[All Fields])) AND ((“infection control”[MeSH Terms] OR (“infection”[All Fields] AND “control”[All Fields]) OR “infection control”[All Fields]) AND (“weights and measures”[MeSH Terms] OR (“weights”[All Fields] AND “measures”[All Fields]) OR “weights and measures”[All Fields] OR “measures”[All Fields]))) AND (“South Africa”[MeSH Terms] OR (“South”[All Fields] AND “Africa”[All Fields]) OR “South Africa”[All Fields]).

The search yielded 28 results that were evaluated for relevance to the study. The policy documents and literature on MDR-TB and infection control were analysed thematically to identify the changes that occurred and the factors driving them. We also searched the reference lists of articles identified by the search strategy and selected the ones regarded as relevant. Grey literature in the form of reports, working papers, government documents, white papers and evaluations was used to inform the study.

## Results

### Policy changes on infection control in South Africa

The National Department of Health in South Africa introduced a policy framework on the management of MDR-TB in 1999 [29]. The policy framework emphasised prevention as the key to effective control of MDR-TB, particularly first-line TB treatment as prevention, in order to reduce the risk of first episodes of active TB occurring or recurring in people either exposed to infection or with latent TB. One of the strategies involved hospitalisation of patients for several months until three consecutive monthly sputa were culture negative. The policy stipulated the provision of special and well-ventilated wards in existing hospitals as a cost-effective way of managing the spread of MDR strains. If there was no negative pressure ward, MDR-TB patients were to be treated in wards with the doors closed and the windows open. In addition, sputum collection was to take place in the open air and on the sunny side of the ward. A special glass roofed veranda, open to the outside was to be built for this purpose. Inside the ward, it was mandatory for ward staff to wear particulate respirator masks impermeable to droplet nuclei. Patients were to wear ordinary masks to prevent an explosive spread. Other environmental requirements included installation of extraction fans.

As a result of increasing incidence of and deaths due to MDR-TB, the South African government enacted the South African National Tuberculosis practical guidelines in 2004 [30]. The failure to control the spread of MDR-TB was noted as a serious threat to individual patients as well as to communities. The policy reiterated the IPC measures of the previous policy and went further to provide more guidelines. For example, the need for enough windows to allow for more ventilation was emphasised; they were to be opened to the outside and not to other wards and, where possible, windows were to be on opposite sides of the room to allow for cross ventilation. Doors were to be kept open to maximise ventilation if they did not open to other wards or rooms. In areas where maximum natural ventilation was not possible, overhead fans were to be installed to enhance ventilation. Mechanical ventilation was recommended in areas where there was a high concentration of infectious droplets to promote air entry into the room and extraction from the room to the outside. Furthermore, the guidelines stipulated the need to use exhaust ventilation systems that allowed for exchange of air in the room as well as extraction of air to the outside. They made a provision for negative pressure ventilation, with the room to be kept at negative pressure to the outside, thus ensuring that air was drawn into the room and exhausted directly to the outside. Ultraviolet germicidal irradiation was suggested as an adjunctive measure. The implementation of these measures was to be guided by the assessment of risk as well as the availability of resources. Despite the additional guidelines, cases of MDR-TB continued to increase [31].

A policy on the management of drug-resistant TB was developed in 2010 and incorporated IPC measures for MDR-TB [32]. The policy provided strategies for IPC and occupational health services for patients and healthcare workers. It was driven by the notion that the management of MDR-TB was an evolving strategy, hence the need for adaption through evidence-based information. The major highlight of the policy was that the management of MDR-TB would be conducted in an environment with appropriate IPC measures to prevent nosocomial transmission of drug-resistant TB. In addition, the policy designated infection control officers and committees with the responsibility of developing and monitoring infection control plans on a regular basis to ensure the effectiveness of the interventions implemented.

In 2011, a policy framework was enacted on decentralised and deinstitutionalised management of MDR-TB for South Africa [33]. It made radical changes, as it was clear from previous experience that prolonged admission of MDR-TB patients in specialised hospitals was not feasible. It was noted that the number of patients diagnosed with MDR-TB exceeded the number of available beds per province. It was expected that the number of patients would continue to rise and that would result in the waiting list growing. For example, of approximately 9070 cases of MDR-TB notified in 2009, fewer than 5000 were started on treatment in the nine provinces of South Africa. Based on the growing evidence, the policy emphasised the need for improving the control of MDR-TB through the decentralisation of services. The policy provided five levels of care with admissions either into a central provincial MDR-TB unit or a decentralised unit with patients being referred to satellite MDR-TB units closer to their homes, home-based services or community support services as their condition stabilised [34].

The policy stipulated that each province, depending on need, would have a number of decentralised MDR-TB units. These units were meant for the initiation and management of MDR-TB patients in a defined geographical area, initially as inpatients, but then as outpatients. These units could be whole hospitals or wards or sections of existing provincial, district or sub-district level hospitals. Patients diagnosed with MDR-TB who were smear microscopy positive were to be hospitalised for a period of 8 weeks or until they became smear negative on two consecutive tests. The decentralisation process brought an urgent need for additional and appropriate infrastructure for MDR-TB. Given the limited facilities, the policy did not make it mandatory for MDR-TB patients to be admitted to hospital for the duration of treatment. Instead, the policy recommended home IPC measures, stipulating adequate ventilation/open windows, isolating patients (own bedroom where possible), promoting cough hygiene and ensuring that patients used surgical masks during waking hours while at home or when meeting with others. Patients were to refrain from close contact with children and maximise time in an open-air environment.

The policy on decentralisation in the treatment of MDR-TB was aligned with the WHO recommendation for National Tuberculosis Control programmes for health system strengthening, mainly through the process of transferring service delivery to manageable units at various levels, including the community [35]. Decentralisation of diagnostic and treatment services of MDR-TB was considered a means to improve access for all patients, especially for those from low-resource settings [36]. The policy framework was also driven by evidence that decentralised MDR-TB facilities provided more effective IPC measures that took social and family pressures into consideration [33]. A study on the treatment for MDR-TB at a specialised TB treatment centre in KwaZulu-Natal revealed delayed initiation of treatment as a challenge that emanated from centralisation [37]. Prolonged hospitalisation also had socioeconomic impacts as some patients were forced to relinquish work and home responsibilities. It was noted that a significant proportion of MDR-TB was due to ongoing transmission of already resistant strains [33]. As a result, emphasis was given to the importance of a decentralised care model in improving efficiency and effectiveness for MDR-TB patients.

Despite the policy on decentralisation and deinstitutionalisation, many MDR-TB patients were hospitalised to receive treatment. This invoked the need for IPC measures in healthcare facilities, particularly given that much nosocomial transmission was likely due to undiagnosed and untreated MDR-TB patients [17]. In 2013, an updated policy on drug-resistant TB was published with specific guidelines on the management of MDR-TB. The policy indicated that the management of patients would be conducted in dedicated MDR-TB units, in other healthcare facilities and in the community by trained healthcare workers [38]. The policy stipulated the need for multidisciplinary clinical management teams in all MDR-TB hospitals. The teams consisted of infection control officers and committees who had the mandate of conducting risk assessments. Suspected, but unconfirmed, MDR-TB patients were to be isolated in a well-ventilated side ward in a district hospital, if space allowed. If at home, they were to be educated about cough hygiene and IPC measures.

As control of MDR-TB was proving to be difficult in South Africa, various studies were conducted with the aim of finding effective ways to prevent the spread of the disease. The studies informed the evolution of MDR-TB IPC measures resulting in evidence-based policymaking.

### Evidence from research

As early as 2002, just a year before TB was declared an emergency in South Africa, the WHO estimated that South Africa had over 14,000 cases of MDR-TB every year, with the country ranking among the top ten countries in the world with the highest number of cases [9, 39]. Various studies revealed that the treatment of MDR-TB in South Africa was not sufficient unless backed up by IPC measures [13, 40, 41]. A study by Ghandi et al. [17] indicated that MDR-TB treatment required a longer course, is more toxic and is more costly than first-line treatment of TB, and is not readily available in resource-limited settings. The challenges posed by MDR-TB, particularly its transmission in the community and healthcare settings, impeded the implementation of antiretroviral scale-up programmes, which were aimed at controlling the HIV epidemic, decreasing TB-related mortality and reducing the incidence of TB and MDR-TB [17, 41]. Research suggested that nosocomial transmission and previous hospitalisation were strong risk factors for MDR- and XDR-TB [8, 17]. A study by Bamford and Taljaard [42] suggested that the hospitals could be breeding grounds for MDR-TB. Another study by Gandhi [43] in Tugela Ferry linked the outbreak of the epidemic to poor airborne infection control.

The outbreak of drug-resistant TB in 2006 in a rural hospital in KwaZulu-Natal, where 39% of 185 culture-confirmed patients had MDR-TB, displayed the severity of the problem [9]. Basu et al. [44] predicted that, without new interventions such as IPC measures, the proportion of inpatients with any form of MDR-TB would increase from 51% in 2007 to 78% in 2012. The combination of a large population of HIV-infected susceptible hosts with poor TB treatment success rates, a lack of airborne infection control, limited drug-resistance testing, and an overburdened MDR-TB treatment programme provided ideal conditions for an MDR-TB epidemic of unparalleled magnitude [8].

The overwhelming challenge of MDR-TB in South Africa, contextualised by high prevalence of HIV infection, threatened to consume public health programmes and undermine the success of antiretroviral therapy rollouts [45]. Research by Dheda et al. [46] revealed that many patients with MDR-TB did not only have poor treatment outcomes, but also had high-grade resistance and continued to have positive cultures despite treatment intervention. The problem presented acute ethical dilemmas on whether or not MDR-TB patients should be discharged into their communities; the major concerns were whether treatment was to be suspended to prevent further acquisition of resistance or whether patients should be isolated from society, to which they posed a threat. Although discharge of infectious and incurable patients back into the community was criticised, the question that arose was whether there were any alternatives in resource-poor settings [21]. Such questions revealed the complexity of the problem of MDR-TB in South Africa. Andrew et al. [45] found that, in a sample of 17 patients who developed MDR- or XDR-TB while being treated for a less resistant form of TB, all cases were due to exogenous reinfection. The study thus highlights exogenous reinfection as an important mechanism for the development of MDR-TB in patients who are on TB treatment.

The policy on integrated, home-based treatment was thus informed by research findings revealing that treatment outcomes for MDR-TB in South Africa were poor as centralised, in-patient treatment programmes struggled to cope with rising prevalence and HIV co-infection. There was no evidence that hospitalisation actually limited community transmission, and it was likely that most patients had been infectious for several months before hospitalisation given the delays in diagnosis and treatment under routine programme conditions in South Africa [47]. A 2008–2010 study involving a sample of 80 MDR-TB patients to assess the model of decentralised treatment in South Africa revealed that retention rates were high, as only 5% of patients defaulted, while the preliminary outcomes were favourable, with 77% cured/still on treatment and few having had severe adverse events (8%) or having died (6%) [48]. The research findings pointed to home-based treatment for MDR-TB as a good model to expand capacity and achieve improved outcomes in rural, resource-poor and high HIV-prevalent settings. Linked to this, the WHO endorsed the Stop TB Strategy, an approach that was aimed to reduce the burden of MDR-TB in line with global targets set for 2015 with emphasis on strengthening infection control in health services, other congregate settings and households [49].

As nosocomial infection of MDR-TB continued to increase in healthcare settings, the Council for Scientific and Industrial Research worked towards developing a management tool for airborne infection control that would identify critical control points where the risk of transmission to patients and staff was particularly high. The project revealed the need to design and build a public interface of health facilities, such as outpatient areas and casualty wards, according to clinically acceptable standards in order to provide the essential requirements for infection control [50]. Consequently, the Council for Scientific and Industrial Research was designated the task by the National Department of Health to research and facilitate the design and construction of new long-term accommodation units for 400 patients in nine centres across the country [51]. The project provided an opportunity to review the existing policy, develop guidelines for long-term accommodation of patients with drug-resistant TB and research, and test and validate the performance of accommodation units provided through the project. This culminated in national building regulations and standards for healthcare facilities which stipulated that, where possible, patients were to be accommodated in single rooms with en-suite facilities, a staff zone (nursing station and support rooms) was to be physically separated from the patients’ infectious zone, and natural ventilation was to be prioritised over artificial ventilation in all patient areas to achieve maximum air changes at all times [52]. The shift from artificial ventilation towards natural ventilation resulted from the former being limited by engineering constraints and costs such as the increasing risk of power outages and continuous escalation in electricity tariffs, while the latter, despite its reliance on climatic conditions, had benefits such as low cost of installation, operation and maintenance [50, 52].

## Discussion

The policy changes with regard to IPC measures for drug-resistant TB in South Africa can be explained, in terms of policy streams theory, as an interplay of the problem of the disease, the political forces that prevailed and alternative proposals that were available. In the first instance, the recognition of MDR-TB as a social and medical problem in South Africa was the major driving factor for the policy changes. Indicators in the form of numbers of MDR-TB cases showed continued growth and meant an increasing burden of the disease on both the government and society.

The magnitude of the problem of TB in general and MDR-TB in particular prompted policy changes on infection control as the incidence of MDR-TB continued to increase. The magnitude of the problem came under the spotlight when TB was declared a national emergency in 2003 [1] and a national crisis in 2005 [53]. The declarations served as focusing events, which according to Kingdon [26], propel issues to the top of the policy agenda. Despite the enactment of a dedicated MDR-TB policy in 2000, the incidence and case–fatality rates continued to increase [49]. This meant that concerted efforts were needed towards comprehensive and programmatic management of MDR-TB. The impetus was towards making changes to the existing policy as it had failed to yield positive results. According to Weyer [54], most public health settings lacked adequate and appropriate infection-control measures; this was juxtaposed with an extremely high prevalence of HIV (both in patients and healthcare workers), which represented a public health emergency requiring segregation of infectious patients, urgent improvements in IPC measures and a rapid, appropriate response to outbreaks. The need for policy change was reinforced by the outbreak of XDR-TB in KwaZulu-Natal from 2005 to 2006, which served as a serious warning that gains made in the treatment of TB could be lost if IPC measures for MDR-TB were not effectively and rapidly addressed [17]. This demanded radical change in infection control resulting in the 2010 policy framework, which called for decentralisation of MDR-TB services.

Thus, as a result of the increasing problem of MDR-TB, policy proposals were generated to change the status quo. As indicated by Kingdon [28], an increasing challenge with no immediate solution compels a community of specialists to formulate proposals to address the situation. That was the case with MDR-TB in South Africa as stakeholders, such as the Department of Health, non-governmental organisations and policymakers, sought to find alternative policy frameworks in the wake of the increased incidence over the period studied. The challenge with infection control for MDR-TB revolved around inadequate accommodation in public hospitals. It was difficult to comply with infection control strategies in the crowded settings of public health facilities in South Africa. Crowding posed a health hazard to healthcare workers (nurses and medical care officers). A cross-sectional descriptive study conducted from June to September 2009 in 24 hospitals for drug-resistant TB across South Africa [55], revealed that the greatest fear of healthcare personnel working in drug-resistant TB wards was contracting MDR- or XDR-TB and infecting others. Such fear could negatively impact the provision of quality patient-centred care. An investigation into the personal experiences and attitudes of medical doctors towards TB showed that the majority were concerned by the lack of infection control strategies at the workplace [56]. Such findings further amplified the need for improved and effective IPC measures.

Various policy proposals were put forward, but their feasibility was determined by the availability of resources, particularly in the form of adequate infrastructure [57, 58]. According to Kingdon [28], not all the proposals have the propensity to rise to the policy agenda as some are discarded due to the costs involved during implementation. In the case of infection control, priority was given to policy proposals that were technically feasible, not too expensive and acceptable to the mass public. Policy proposals that required substantial resources for implementation took time to be adopted – it was not until 2010 when the policy on decentralisation was adopted after financial resources were provided by the Global Fund [34]. The notion of decentralisation of health services in general dates back to the publication for comment on an enabling policy for the development of a District Health System in South Africa [59], which emphasised the need for devolving decision-making to the lowest appropriate level.

The overwhelming number of MDR-TB patients in South Africa facilitated the policy changes towards decentralisation and home-based care. The policy framework on decentralised and deinstitutionalised management of MDR-TB in South Africa was driven by the lack of hospital beds and the successful demonstration of decentralisation in pilot sites [33]. The high incidence of MDR-TB cases resulted in severe congestion of hospitals as they failed to cope with the number of patients [8]. The lack of resources in terms of adequate and well-ventilated MDR-TB wards to admit patients prompted policy change towards community-based care. On the other hand, the policy change towards community-based care was also driven by the dissatisfaction of MDR-TB patients with the lengthy period of hospitalisation. For example, patients in Eastern Cape staged a demonstration in December 2009 demanding free passes to go to their homes; this act of civil disobedience came after a group of MDR-TB patients at Jose Pearson Hospital in Port Elizabeth escaped by cutting through the hospital’s perimeter fence [60].

It is impossible to separate the transmission of TB from the spread of HIV in the South African context. TB is the most common cause of death in AIDS patients, and the most common opportunistic infection for HIV-infected patients. The politics concerning the response of the South African government in addressing the HIV/AIDS epidemic filters through in the incidences of all forms of TB. Karim et al. [9] argue that apartheid created social, economic and environmental conditions that were favourable for the transmission of TB. These include overcrowded informal settlements, the migrant labour system and a deliberately underdeveloped healthcare service for black people. Given the historical background of disease in the apartheid era, there were high expectations that the new government would make a concerted effort towards addressing the disease. However, there was rapid spread of HIV during 1995–2000, followed by a phase from 2000 where the AIDS mortality rates increased, resulting in smaller increases in HIV prevalence [9, 61].

The fight against HIV/AIDS was already recognised in the Mandela administration, with the adoption of the Networking HIV/AIDS Community of South Africa (NACOSA AIDS) plan, and was prioritised as one of the African National Congress’s Reconstruction and Development programmes [62, 63]. However, it was never given high profile political support. The Mbeki government’s stance that HIV does not necessarily cause AIDS, and the then Minister of Health’s strategy of healthy living to tackle the HIV/AIDS epidemic, were highly controversial in South Africa. It was only in 2004 that the first National Strategic Plan against HIV came into effect, resulting in antiretroviral therapy being made available to the public. However, during Mbeki’s administration, progress remained slow. For example, it is estimated that 330,000 lives could have been saved if the decision to provide antiretroviral therapy had been made 3 years earlier [9]. The change in administration in 2008, followed by the 2009 elections and a new Minister of Health, resulted in HIV becoming the top public health priority with a rapid increase in testing and provision of antiretroviral therapy. For example, the number of functional antiretroviral therapy clinics increased from 362 in 2008 to 3000 in 2013, while the number of people receiving antiretroviral therapy increased to about 2.5 million in late 2013, with a target of 3.1 million people in 2015 [64].

Civil society and patient organisations played a crucial role in the policy changes for MDR-TB. The experience with HIV in South Africa was instructive as strong civil society action spurred policy changes and raised awareness of entitlements to treatment among those affected by the pandemic [65]. Increasing discontent over the failure by the government to control the disease was triggered by the continuous rise in new cases of the disease due to poor treatment outcomes. South Africans, particularly the black majority, became frustrated with the increasing cases of TB infection and blamed the government for its failure to control the disease [7, 66]. Disappointed by the failure of the South African government to control MDR-TB, activist organisations, namely the Treatment Action Campaign and the TB/HIV Care Association, marched to Cape Town’s Parliament buildings on March 24, 2009. They handed over a petition on TB, which demanded that the government invest more time, energy and money in developing strategies to tackle TB [67]. Such pressure from activists on the government contributed to the policy changes towards controlling the spread of MDR-TB.

The South African National Tuberculosis Association used its network of branches and care groups across South Africa to influence policy changes in partnership with other stakeholders. For example, along with patient organisations, such as the Friends of the Sick Association, it provided supportive, preventive and curative services to TB patients and their families, thereby demonstrating the feasibility of decentralised care [9]. This was important in promoting shifts in policy, particularly from centralised to decentralised community-based treatment of MDR-TB.

The mass media contributed to setting the agenda for the management of MDR-TB. By drawing and sustaining public attention on the challenges posed by MDR-TB in South Africa, the media served as a conduit between the government and the public, a unifier of diverse viewpoints and a national watchdog that reflected and shaped the public discourse. For example, a study on the representations of MDR- and XDR-TB in 310 South African newspapers from February 2004 to July 2009 revealed three main themes, namely patient-centred causes (32.6%), lack of infection control procedures (18.7%) and health systems failures, while the solutions to tackling the disease focused on patient-targeted solutions (38.4%), improving infection control (12.3%), systems restructuring (10.6%) and diagnostic and therapeutic options (10%) [57]. What is explicit from the media coverage is the emphasis on ICP procedures as both a cause of and a solution to MDR- and XDR-TB. Thus, the media played a role in problem definition, causal interpretation and policy recommendation.

International humanitarian medical organisations also contributed towards policy changes of MDR-TB in South Africa through advocating for decentralisation of service delivery. Médecins Sans Frontières conducted pilot studies in Khayelitsha to pre-test the efficacy of a decentralised model of care in which clinically stable patients with drug-resistant TB were diagnosed and managed by clinicians in facilities at a primary healthcare level [68]. The interim outcomes were presented in reports in 2009 [69] and 2011 [70], and provided useful insights into successes and areas for further improvement. The pilot program served as a feasibility study and experimental trial. Following remarkable achievements in the use of the decentralised model, such as increased case detection, strengthened patient support, increased treatment initiation rates, decreased time from diagnosis to treatment initiation, improved IPC measures and more efficacious treatment regimens, the management and responsibility of the routine, decentralised drug-resistant TB programme was handed over to the Department of Health at the end of 2013 [71]. The potential benefits of a new policy are often subject to debate and hard for governments to predict. The pilot initiative by Médecins Sans Frontières provided first-hand information and confirmed the benefits of the model [68], thereby paving way for the adoption and subsequent implementation of the policy.

Policy changes were also strongly grounded in evidence from research. For example, a pilot study that was conducted in the Hlabisa sub-district in Kwazulu-Natal revealed that community-based treatment for MDR-TB was both feasible and safe in rural South Africa and could be implemented within the existing TB control programme in rural areas [47]. The policy on integrated, home-based treatment was based on empirical evidence that centralised and in-patient treatment programmes were not effective in controlling MDR-TB in South Africa [33]. This was complemented by studies that showed no evidence to substantiate the claims that hospitalisation of MDR-TB patients reduced transmission of the disease to the community [47, 72]. Instead, various studies showed that the hospitalisation of MDR-TB patients in crowded and resource-limited settings posed risks for nosocomial transmission [45, 73–75]. A mathematical modelling study based on the transmission of drug-resistant TB in South Africa suggested that a large proportion of newly transmitted cases could be averted through a combination of community-based care and simple mask wearing [44, 76]. A study that was conducted in South Africa to evaluate the use of simple surgical masks on MDR-TB revealed a 56% decreased risk of TB transmission [77]. Cox et al. [78] assessed a novel way of improving ventilation for TB infection control in health facilities by conducting a study in primary care clinic rooms in Khayelitsha. The study revealed that natural ventilation can be increased through the use of wind-driven roof turbines, which are a simple and low cost technology requiring less maintenance and no electricity as they are driven by natural forces. Such findings informed the evolution of MDR-TB infection control policies as they underscored the importance of implementing effective airborne IPC measures in the fight against the disease.

## Conclusion

The growing incidence of MDR-TB in South Africa propelled the problem to the top of the policy agenda. The increase in the incidence of the disease against a background of growing levels of dissatisfaction among patients, the shortage of physical, human and financial resources in public hospitals, and the different ideologies of the political leadership motivated policy changes. Prevention of MDR-TB was considered a priority for MDR-TB control, given the need to limit the spread of the disease and considering the high cost, toxicity and poor treatment outcomes with available therapies. The changes inevitably arose due to the mounting pressure that was exerted by the different stakeholders who felt that the status quo was unsustainable. The high incidence of MDR-TB served as a signal that the health system was failing to adequately deal with the problem of TB. The need to address the challenges in controlling MDR-TB opened policy windows filled with alternative proposals and resulting in policy changes. The policy changes were influenced by the context of the problem of MDR-TB in South Africa. The factors that drove changes in MDR-TB infection control policies in South Africa are linked to the context in which the disease was embedded. We conclude that policy changes for infection control of MDR-TB in South Africa were critically influenced from a plethora of different sources rooted in the socioeconomic and political environment, and were supported by research evidence. The policy changes can be framed, using Kingdon’s policy streams approach, as an interplay of the problem of the disease, the political forces that prevailed and the alternative proposals available.

## Abbreviations

IPC: Infection prevention and control
MDR: Multidrug-resistant
TB: Tuberculosis
XDR: Extensively drug-resistant.
